# Possible Enzymatic Downregulation of the Natriuretic Peptide System in Patients with Reduced Systolic Function and Heart Failure: A Pilot Study

**DOI:** 10.1155/2018/7279036

**Published:** 2018-07-26

**Authors:** Syed S. Zaidi, Ryan D. Ward, Kodangudi Ramanathan, Xinhua Yu, Inna P. Gladysheva, Guy L. Reed

**Affiliations:** ^1^Department of Medicine, University of Tennessee Health Science Center, College of Medicine, 956 Court Ave, Memphis, TN 38163, USA; ^2^Veterans Administration Medical Center, Memphis, TN 38163, USA; ^3^School of Public Health, University of Memphis, Memphis, TN 38152, USA; ^4^Department of Medicine, University of Arizona, College of Medicine-Phoenix, Phoenix, Arizona, USA

## Abstract

**Background:**

In patients with reduced systolic function, the natriuretic peptide system affects heart failure (HF) progression, but the expression of key activating (corin) and degrading enzymes (neprilysin) is not well understood.

**Methods and Results:**

This pilot study (n=48) compared plasma levels of corin, neprilysin, ANP, BNP, and cGMP in control patients with normal ejection fractions (mean EF 63 ± 3%) versus patients with systolic dysfunction, with (EF 24 ± 8%) and without (EF 27 ± 7%) decompensated HF (dHF), as defined by Framingham and BNP criteria. Mean ages, use of beta blockers, and ACE-inhibitors-angiotensin receptor blockers were similar between the groups. Corin levels were depressed in systolic dysfunction patients (797 ± 346 pg/ml) versus controls (1188 ± 549, p<0.02), but levels were not affected by dHF (p=0.77). In contrast, levels of neprilysin (p<0.01), cGMP (p<0.001), and ANP (p<0.001) were higher in systolic dysfunction patients than controls and were the highest in patients with dHF.

**Conclusions:**

Levels of neprilysin, ANP, BNP, and cGMP increased in patients with reduced systolic function and were the highest in dHF patients. Conversely, corin levels were low in patients with reduced EF with or without dHF. This pattern suggests possible enzymatic downregulation of natriuretic peptide activity in patients with reduced EF, which may have diagnostic and prognostic implications.

## 1. Introduction

The natriuretic peptide system plays a central role in heart failure (HF) and members of this pathway are the target for therapeutic (neprilysin) and diagnostic (ANP, BNP) strategies. The natriuretic peptide system is controlled in part by enzymes such as neprilysin (membrane metalloendopeptidase, neutral endopeptidase) and corin, a transmembrane serine protease expressed by cardiomyocytes. Neprilysin cleaves and inactivates natriuretic peptides and promotes HF [1]. In contrast, corin activates pronatriuretic peptides and opposes the development of experimental HF [2–6]. Corin and neprilysin can be released from the cell surface into circulation and detected in plasma or serum like many other membrane-bound proteases [3, 7, 8]. Plasma corin levels are reduced in patients with decompensated (d) HF with reduced (r) ejection fraction (EF) leading to diminished pronatriuretic peptide activation [7, 9]. Recent experimental data suggests that corin cardiac expression and plasma levels may reflect the severity of cardiomyopathy and impaired systolic function and precede the development of dHF [2]. In this pilot study, we examined whether corin and neprilysin plasma levels are altered by the presence of reduced systolic function in patients with and without dHF.

## 2. Material and Methods

### 2.1. Study Design

The study protocol and consent form were approved by the IRB of the Memphis VA Medical. All participants gave written informed consent to participate in the study. The investigation conforms with the principles outlined in the Declaration of Helsinki 1964. In this prospective study, patients (ages 50-70) admitted to the medical services or from the HF clinic were eligible for the study if they provided informed consent. Clinical data and samples were collected from 3 groups of patients: no HF and normal EF (nEF), no HF and systolic dysfunction (rEF≤ 50%), and HF with systolic dysfunction (rEF≤ 50%). Clinical dHF was defined by abbreviated Framingham criteria using a HF scoring system that scores the presence of orthopnea, paroxysmal nocturnal dyspnea, exercise tolerance, sinus tachycardia, jugular venous pressure, hepatojugular reflex, third heart sound, basal crackles, hepatomegaly, and peripheral edema and by the presence of a BNP > 400 pg/ml [10, 11]. Consistent with these criteria, patients with clinical dHF had a sore of two or more. All patients had an echocardiogram or other measurement of their ejection fraction (EF) done within 60 days of enrollment.

A detailed medical history was obtained from the patient. Other pertinent clinical data were obtained from the computerized patient record system. Patients were excluded who had conditions that might independently affect the natriuretic system such as chronic kidney disease (estimated glomerular filtration rate <60 ml/min/1.73 m^2^), pulmonary hypertension (pulmonary artery pressure >55 mmHg by transthoracic echocardiography), myocardial infarction within the last 6 weeks, critical valvular heart disease, metastatic or terminal cancer, morbid obesity (BMI > 35), and cardiopulmonary support.

### 2.2. Blood Sample Collection

Venous blood samples were collected using standard EDTA-aprotinin tubes and immediately stored in ice. The blood samples were centrifuged at 3000xg for 20 min at 4°C and plasma aliquots were stored at −80°C until analysis [7].

### 2.3. Heart Failure Biomarkers Measurement

ANP was measured by an enzyme immunoassays (ELISA) using antibodies against the N-terminal sequence (Phoenix Pharm, Inc.). Corin levels were measured in plasma as we have described using DuoSet ELISA development assay (R&D Systems.) [7]. cGMP and neprilysin were measured in plasma by ELISA according to the manufacturer's protocols (R&D Systems, Thermo-Fisher Scientific). Other clinical chemistries were measured by the standard clinical laboratory methods in the hospital.

### 2.4. Statistical Analysis

The sample size was selected based on our previous study to provide >90% statistical power to detect a simultaneous difference in corin, ANP, cGMP, and neprilysin levels between patients with nEF and patients with rEF and with or without dHF [7]. Unless otherwise indicated, continuous data are presented in tabular form or in box plots indicating the medians with upper and lower quartile values. Normally distributed data were analyzed by Student's* t*-test. Corin data were subjected to a square root transformation based on Box-Cox methodology. Multivariate regression was performed to examine whether other variables affect the association between corin and EF. A two-tailed* p* value < 0.05 was considered statistically significant.

## 3. Results and Discussion

Three patient groups were enrolled from the Memphis VA medical service or HF clinic: controls with normal EF (nEF, ≥50%) and no dHF and reduced EF (rEF<50%) with and without dHF. DHF was prospectively defined by Framingham criteria for objective signs of fluid retention using a standardized scoring system (see Table 1 of ref. [10]) and by a BNP>400 pg/ml. The demographic information for each group is shown in Table 1. All patients were male, reflecting the composition of the VA. There were no significant differences in average age (59-61 years) among the three groups. The proportion of African-Americans was significantly higher in the rEF+dHF patients than controls (81% versus 31%, p<0.05) or than rEF patients (25%). The mean BMI for all groups was in the overweight category, but it was lower in the rEF+dHF group than in the control or rEF patients. A history of coronary artery disease was more common in control patients (56%, p<0.05) or rEF patients (63%, p<0.05) than in those with rEF+dHF (13%). A history of hyperlipidemia was more common in patients with rEF than those with rEF+dHF (p<0.05). There were no significant differences between the groups in the frequency of other existing medical conditions (Table 1).

The laboratory data for each group is shown in Table 2. Triglycerides and albumin were significantly lower in patients with rEF+dHF as compared with rEF patients, while alkaline phosphatase and total bilirubin were significantly higher indicating liver dysfunction, which is frequent at HF [12]. Total cholesterol was lower and total bilirubin was higher in rEF+dHF patients than in controls.

The mean EFs (±SD) in controls were 63 ± 3%. Among subjects with rEFs, there was no significant difference between those with rEF alone (EF 27 ± 7%) and those with rEF+dHF (EF 24 ± 8%). Patients with rEF+dHF differed from control patients in nearly every echocardiographic parameter (Table 3). Mean LV dimensions (LVIDd =6.2, LVIDs=5.4 cm) were larger in the rEF groups versus controls (p<0.001), but there were no significant differences between the rEF and rEF+dHF groups (p=0.88-0.92). There were no significant differences in left ventricular mass between the controls (225 ± 82 g), rEF (343 ± 213 g), or rEF+dHF (290 ± 180 g; p= 0.27).

Radiographic evidence of HF was present in 88% of patients with rEF+dHF, but this was not seen in the other groups. Most patients with rEF were prescribed beta blockers and ACE-I/ARBs (Table 4); the frequency did not significantly differ between the rEF, rEF+dHF, and control groups. Diuretic use was more common in patients with rEF+dHF than rEF patients (88% versus 56%, p≤0.05) and it was also more frequent in rEF+dHF patients than in control patients (88% versus 38%, p<0.01). Spironolactone, the only mineralocorticoid antagonist used, was significantly more common in patients with rEF (56%, p<0.01) and rEF+dHF (50%, p<0.05), than in control patients (13%). Similarly, digoxin use was more common in patients with rEF (31%, p<0.05) than in control patients (0%, p<0.05). Statin use was more common in patients with nEF or rEF than in patients with rEF+dHF (p<0.05).

**Figure 1:**
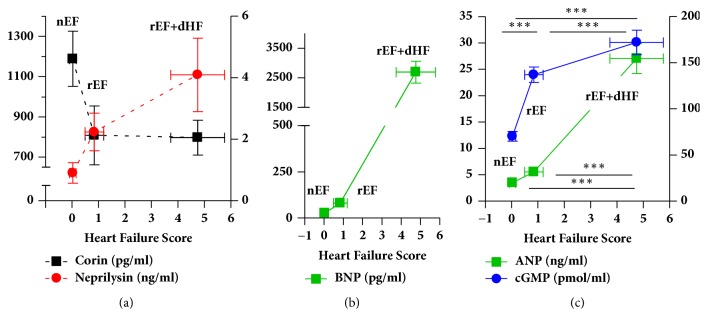
**Corin, neprilysin, BNP, ANP, and cGMP levels in patients with normal (nEF) and reduced (rEF) ejection fractions, with and without decompensated (d) HF.** (a) Corin and neprilysin plasma levels (mean±SE) in patient groups by corresponding HF score (mean±SE). (b) BNP levels (mean±SE) in groups with nEF (control), rEF, and rEF+ dHF according to HF score, where dHF is defined by a HF score ≥ 2 [10]. (c) ANP and cGMP plasma levels (mean±SD) in patient groups by HF score (mean±SE). N= 48, 16 per group. ^*∗∗∗*^*p*< 0.001, one-way ANOVA.

ELISA was used to measure corin, N-ANP, neprilysin, and cGMP levels in plasma samples collected in EDTA-aprotinin tubes to prevent coagulation and proteolysis. The assay ranges for these natriuretic system proteins were corin (linear detectable range 30-1000 pg/ml), neprilysin (linear detection range 0.2-16 ng/ml), ANP (detectable range 0-100 ng/ml; linear range 0.1-10 ng/ml), and cGMP (linear detectable range 2-500 pmole/ml). Plasma samples were diluted 2-10-fold to fit the calibration curves/detectable assay ranges. All other measurements including BNP (Siemens Centaur) were by the VA clinical laboratory. Median corin levels were significantly lower in all patients with rEF, than in controls (p<0.05). In contrast, median neprilysin levels were significantly higher in all patients with rEF than in controls (p<0.01). As HF scores worsened, neprilysin levels appeared to increase (Figure 1(a)). In contrast, corin levels were reduced in patients with rEF but did not appear to change with increasing HF score (Figure 1(a)). Patients with rEF+dHF had higher BNP levels and HF scores than rEF alone or control patients (Figure 1(b)), reflecting clinical dHF, as expected. ANP levels rose significantly with increasing HF (Figure 1(c)). In parallel, there was a significant increase in cGMP levels with more severe HF scores (Figure 1(c)). As neprilysin levels rose, log ANP levels also increased, reflecting a positive correlation between the two measures (r_p_=0.45, p<0.01). However, there was a negative correlation between log corin and neprilysin levels, indicating that, as corin decreased, neprilysin levels rose (r_p_=-0.49, p<0.001).

Recent studies have shown that soluble levels of corin are depressed in patients with HF [7, 9, 13, 14]. Experimental data indicate that cardiac corin expression and plasma levels may fall early in the course of cardiomyopathy, reflecting systolic dysfunction prior to the development of dHF, as indicated by fluid retention and increases in blood levels of ANP [2]. Previous clinical studies have correlated corin levels with functional capacity as assessed by NYHA class, but the present data provide the first evidence in humans, consistent with experimental observations in mice, that depressed plasma corin levels may indicate systolic dysfunction, even in the absence of clinical dHF [2, 13]. By comparison, levels of neprilysin rose in patients with rEF and progressively with increasing HF score. Neprilysin showed a similar pattern to ANP and cGMP levels, which significantly increased in parallel with the severity of HF. In contrast, corin levels showed a significant negative correlation with both neprilysin and ANP levels. Since our data are derived from a modest sample of male patients at a single VA hospital, it will be important to confirm the generalizability of these research findings in other studies.

In addition to HF, natriuretic peptide levels are also modulated by body mass index and other conditions such as age, sex, and other illnesses [15]. We found that natriuretic peptide levels were the highest in those with the most severe HF, who also had the lowest BMIs. Although BMI and HF have opposite effects on natriuretic peptide levels, elevated BNP levels in HF patients still are associated with a poor prognosis [15]. The natriuretic peptides are believed to have protective effects in dHF by opposing the action of the renin-angiotensin-aldosterone system [3]. However, the finding that corin levels are low and neprilysin levels are high in patients with rEF may possibly suggest enzymatic downregulation of the natriuretic peptide system; indeed, impaired natriuretic peptide processing has been reported in patients with HF [7, 16]. Corin levels are known to be downregulated by an IRE-1-dependent mechanism under conditions of enhanced ER stress [17]; much less appears to be known about the mechanisms that regulated neprilysin expression. Nevertheless, the prognostic significance of low plasma corin levels and high neprilysin levels is similar. A recent study found that low corin levels were associated with rEF, lower NYHA functional status, increased cardiovascular mortality, and major adverse cardiac events [14]. Recent data also show that elevated neprilysin levels are associated with enhanced mortality in hospitalized HF patients [8]. Combined inhibition of the renin-angiotensin system and neprilysin was shown to be beneficial in chronic HF patients [18].

In conclusion, corin plasma levels are diminished in patients with rEF, with or without dHF. In contrast, in rEF patients, plasma immune-reactive ANP, BNP, cGMP, and neprilysin levels are higher in patients with dHF. Low corin and high neprilysin levels would be expected to enzymatically downregulate the natriuretic peptide pathway and may be harmful in HF. Consistent with that notion, low corin and high neprilysin levels are associated with a poor prognosis. Further insights into the regulation and activity of the natriuretic peptide system may enhance prognostic and therapeutic precision in patients with HF, by identifying patient subsets that may benefit from specific therapeutic modulation of the natriuretic pathway.

## Figures and Tables

**Table 1:** Patient characteristics.

**Patient Characteristics**	**Normal Systolic Function**	**Reduced Systolic Function (Systolic Dysfunction)**	
	**Control**	**No Heart Failure**	**Heart Failure**
Male (N, (%))	16 (100)	16 (100)	16 (100)
Black Race (N, (%))	5 (31)	4 (25)	13 (81)
Age, yrs (mean ± SD)	59.3 ± 6.8	61.2 ± 4.4	59.6 ± 4.2
BMI (mean ± SD)	28.8 ± 2.6	28.4 ± 3.4	25.6 ± 5.1^§,†^
Heart Failure Score (mean ± SD)	0.0 ± 0.1	0.8 ± 0.4^‡^	4.8 ± 1.0^§,†^
Medical History			
Coronary Artery Disease (N, (%))	9 (56)^§^	10 (63)^†^	3 (19)
Myocardial Infarction (N, (%))	2 (13)	2 (13)	0 (0)
Hypertension (N, (%))	15 (94)	13 (81)	15 (94)
Diabetes Mellitus (N, (%))	6 (38)	8 (51)	9 (56)
Cancer (N, (%))	2 (13)	0 (0)	0 (0)
Cirrhosis (N, (%))	0 (0)	0 (0)	0 (0)
Pulmonary Embolism (N, (%))	0 (0)	0 (0)	1 (6)
Obstructive Sleep Apnea (N, (%))	3 (19)	0 (0)	2 (13)
Arrhythmia (N, (%))	1 (6)	5 (31)	4 (25)
Ethanol Consumption (N, (%))	1 (6)	2 (13)	4 (25)
Chronic Obstructive Lung Disease (N, (%))	2 (13)	2 (13)	5 (31)
Hyperlipidemia (N, (%))	10 (67)	15 (94)^†^	7 (44)

*p*<0.05; ^§^reduced systolic function + dHF versus control, ^†^reduced systolic function + dHF versus reduced systolic function no heart failure, and ^‡^reduced systolic function versus normal systolic function.

**Table 2:** Laboratory results.

**Laboratory Test**	**Normal Systolic Function**	**Reduced Systolic Function (Systolic Dysfunction)**	
	**Control**	**No Heart Failure**	**Heart Failure**
BUN (mg/dL)	16 ± 5	19 ± 6	22 ± 9^§^
Creatinine (mg/dL)	1.0 ± 0.2	1.1 ± 0.2	1.1 ± 0.2
eGFR (mL/min/1.73m^2^)	92 ± 22	78 ± 19	89 ± 29
Sodium (mEq/L)	140 ± 3	139 ± 4	138 ± 4
Chloride (mEq/L)	103 ± 4	102 ± 3	101 ± 5
Bicarbonate (mEq/L)	27 ± 4	29 ± 4	28 ± 5
Leukocytes (x10^3^/mm^3^)	5.8 ± 1.3	7.0 ± 2.2	7.4 ± 3.1
Hemoglobin (g/dL)	12.5 ± 1.5	12.7 ± 1.3	12.1 ± 1.8
Hematocrit (%)	37 ± 5	38 ± 4	38 ± 5
Triglycerides (mg/dL)	139 ± 54	173 ± 79	95 ± 46^†^
Total Cholesterol (mg/dL)	169 ± 32	154 ± 43	128 ± 37^§^
HDL (mg/dL)	41 ± 21	38 ± 8	33 ± 14
LDL (mg/dL)	101 ± 28	82 ± 40	78 ± 29
AST (U/L)	45 ± 40	38 ± 12	50± 23
ALT (U/L)	38 ± 22	33 ± 15	37 ± 19
Alkaline Phosphatase (U/L)	94 ± 41	89 ± 21	130 ± 56^†^
Total Bilirubin (mg/dL)	0.6 ± 0.7	0.6 ± 0.4	1.3 ± 0.8^§,†^
Albumin (g/dL)	3.9 ± 0.5	4.2 ± 0.3	3.6 ± 0.6^†^

*p*<0.05; ^§^rEF + HF versus control, ^†^rEF + HF versus rEF, and ^‡^ rEF versus control. Data represent means ± SD unless otherwise indicated.

**Table 3:** Echocardiography parameters.

**Laboratory Test**	**Normal Systolic Function (nEF)**	**Reduced Systolic Function (rEF)**	
	**Control**	**No Heart Failure**	**Heart Failure**
LVIDs, cm	3.3 ± 0.8	5.4 ± 1.2^‡^	5.4 ± 0.9^§^
LVIDd, cm	4.8 ± 0.8	6.2 ± 1.0^‡^	6.2 ± 0.7^§^
Left Atrium, cm	3.8 ± 0.4	3.9 ± 1.1	4.3 ± 0.5^§^
Ejection Fraction, %	63 ± 3	27 ± 7^‡^	24 ± 8^§^
IVSd, cm	1.1 ± 0.2	1.0 ± 0.2	1.0 ± 0.3
AoRoot, cm	3.7 ± 0.3	3.4 ± 0.4	3.3 ± 0.5^§^
LVOT, cm	2.0 ± 0.1	1.9 ± 0.1	2.1 ± 0.1^†^
LVPWd, cm	1.1 ± 0.2	1.0 ± 0.1	1.0 ± 0.3
FS, %	32.7 ± 7.9	14.9 ± 6.0^‡^	13.4 ± 6.3^§^
LVM, g	225 ± 82	343 ± 213	290 ± 180
RVSP, mmHg	26 ± 10	37 ± 7^‡^	44 ± 4^§^
RVWM Abnormality N, (%)	0 (0)	7 (58)^‡^	5 (36)^§^
LVWM Abnormality N, (%)	0 (0)	9 (75^‡^	11 (79)^§^
RV Size Abnormal N, (%)	0 (0)	4 (25)	9 (60)^§^
RV Function Abnormal N, (%)	15 (100)	11 (73)	3 (10)^§,†^
Mitral Regurgitation N, (%)	1 (7)	2 (13)	9 (56)^§,†^
Tricuspid Regurgitation N, (%)	0 (0)	2 (13)	12 (75)^§,†^
Aortic Regurgitation N, (%)	0 (0)	0 (0)	4 (25)

*p*<0.05; ^§^rEF + dHF versus control, ^†^rEF + dHF versus rEF, and ^‡^ rEF versus control. Data represent means ± SD unless otherwise indicated.

**Table 4:** Medication usage.

**Laboratory Test**	**Normal Systolic Function**	**Reduced Systolic Function (Systolic Dysfunction)**	
	**Control **	**No Heart Failure**	**Heart Failure**
ASA (N, **(%))**	14 (88)	11 (69)	13 (81)
Beta Blocker (N, **(%))**	10 (63)	15 (94)	11 (69)
ACE-inhibitor (N, **(%))**	8 (50)	11 (69)	11 (69)
Angiotensin Receptor Blocker (N, **(%))**	1 (6)	3 (19)	2 (13)
Spironolactone (N, **(%))**	2 (13)	9 (56)^‡‡^	8 (50)^§^
Diuretic (N, **(%))**	6 (38)^§§^	9 (56)	14 (88)^§^
Coumadin (N, **(%))**	1 (6)	3 (19)	4 (25)
Nitrates (N, **(%))**	6 (38)	3 (19)	3 (19)
Statin (N, **(%))**	14 (88)^†^	14 (88)^†^	9 (56)
Digoxin (N, **(%))**	0 (0)	5 (31)^‡^	4 (25)
Antiarrhythmic (N, **(%))**	0 (0)	2 (13)	3 (19)

*p*<0.05; ^§^rEF + HF versus control, ^†^rEF + HF versus rEF, and ^‡^ rEF versus control. ^§§^*p*<0.01; rEF + dHF versus control and ^‡‡^ rEF versus control. Data represent means ± SD unless otherwise indicated.

## Data Availability

The data used to support the findings of this study are available from the corresponding author upon request.
